# Why do We Use the Concepts of Adult Anesthesia Pharmacology in Developing Brains? Will It Have an Impact on Outcomes? Challenges in Neuromonitoring and Pharmacology in Pediatric Anesthesia

**DOI:** 10.3390/jcm10102175

**Published:** 2021-05-18

**Authors:** Pablo O. Sepúlveda, Valeria Epulef, Gustavo Campos

**Affiliations:** 1 Hospital Base San José de Osorno, Service Anesthesiology and Pain, Faculty of Medicine, University Austral, Los Lagos 529000, Chile; 2 Department of Surgery, Traumatology and Anesthesiology, Medicine Faculty, Universidad de La Frontera, Temuco 4780000, Chile; valeriaepulef@gmail.com; 3 Hospital Hernán Henriquez Aravena, Temuco 4780000, Chile; 4 Hospital Pediatrico Niño Jesús, Service of Anesthesiology, Córdoba 5500, Argentina; gustavocampos66@gmail.com

**Keywords:** pediatric anesthesia, EEG, TIVA, propofol, neuromaturation, synaptogenesis, neonatology

## Abstract

Background: Pediatric sedation and anesthesia techniques have plenty of difficulties and challenges. Data on the pharmacologic, electroencephalographic, and neurologic response to anesthesia at different brain development times are only partially known. New data in neuroscience, pharmacology, and intraoperative neuromonitoring will impact changing concepts and clinical practice. In this article, we develop a conversation to guide the debate and search for a view more attuned to the updated knowledge in neurodevelopment, electroencephalography, and clinical pharmacology for the anesthesiologic practice in the pediatric population.

## 1. Introduction

A retrospective study with data from 1996 to 2000 showed the impact of multiple surgeries and anesthesia on learning disabilities and the onset of ADHD in children under 3 years of age [1]. This study confirmed the results of an earlier 1986 study on this topic, despite better monitoring and new drugs available. In December 2016, the Food and Drug Administration (FDA) released a Drug Safety Communication warning that the “repeated or lengthy use of general anesthetic in children younger than three years of age or in pregnant women during the final trimester may affect development of children’s brains”.

The American Society of Anesthesia responded to these FDA claims by acknowledging that while there are data on anesthesia toxicity risks in developing brains [2], these are not conclusive in the clinical setting. In their statement, they affirmed: “It is not yet known whether the anesthetic drug or some other factor is responsible for these findings. Rigorous research to further characterize any possible associations is ongoing” [3].

During the last few years, several studies have been developed in an attempt to clarify the undesirable effects of general anesthesia on children. Studies such as the GAS study, PANDA Project, and MASK study [4,5,6] have preliminary results. The GAS study compared regional vs. general anesthesia and concluded that general anesthesia of less than 1 h in young infants does not alter neurodevelopmental outcome at 5 years of age compared to the use of awake regional anesthesia. The other two studies evaluated the use of general anesthetics and the impact on neurodevelopment, and neuropharmacology was not associated with deficits in intelligence quotient; however, the parents of multiply exposed children reported increased problems in terms of executive function, behavior, and reading. All these studies include retrospective or prospective analyses, but they do not differentiate between drug techniques (inhaled or intravenous) and whether electroencephalogram (EEG) brain monitoring was performed or not.

## 2. The Core of the Current Debate

The conceptualization of pediatric anesthesia, particularly the sedative component, as a pharmacological phenomenon associated principally with neocortical activity, presents many conceptual questions and a deficit of objective data in order to interpret whether the anesthetic action fulfills all the desired objectives. For this reason (and others), anesthetics are frequently performed with techniques, drugs, or concepts used in mature brains. For example, the full significance of recent electroencephalographic and functional imaging data obtained in children during anesthetic cortical depression is not yet fully understood, which has uncertain implications for our assessment of current clinical practice. The same applies to pharmacological data.

For many years, it was assumed that young children, especially neonates, had no response to noxious aggression. Just as this concept has been demonstrated to be incorrect, similar concepts are being assumed about pediatric sedation. For example, the full significance of electroencephalographic and functional imaging data obtained in children exposed to anesthetic is not yet fully understood. In a recent study on infants <6 months of age, the relative percentages of density spectral array did not correlate with end-tidal sevoflurane, there being an absence of coherence in alpha and beta frequencies [7]. This also applies to pharmacologic data. These problems create uncertain implications for our assessment of current clinical practice. These conceptual errors have occurred due to the direct transfer of knowledge to pediatric patients from studies of the adult brain. [8,9]

Nociceptive reactivity can be studied from 19 weeks of gestation age. At 20 weeks, the fetus already responds by releasing cortisol and beta-endorphins and noradrenaline, measured in the umbilical cord [10].

Facial expressions of pain appear from a postconceptional age of 28 weeks, and cortical blood flow activity associated with the bilateral somatosensory cortex S2 has been measured between 25 and 45 weeks of gestation after venous puncture. However, this does not ensure that at that age it is possible to develop perceptive abilities, nor to interpret or memorize them as an explicit memory event. Many of these responses are pre-programmed responses of subcortical origin [11,12].

The concept of minimum alveolar concentration (MAC50) is frequently used in children to guide the anesthetic dosage using population statistical data. Difficulties in titrating inhaled drugs to well-defined pharmacodynamic targets are still major challenges. Generally, the use of MAC50 as a pharmacodynamic concept relies on the patient’s immobility as a clinical objective. This concept originates from the observation that much higher doses of an anesthetic agent are needed to inhibit spinal responses than the concentrations needed to achieve patient unconsciousness. However, this results in unnecessary depression of cortical activity to achieve “unconsciousness and immobility” [13].

In pediatric practice, therefore, it is very unusual to titrate (individualize) the anesthetic requirement of the sedation/unconsciousness component. The drug dosages are usually guided by statistical tables, indirect markers such as hemodynamic response, or subcortical responses such as movement to surgical stimuli.

## 3. Neural Development, EEG, and the Emergence of Consciousness

Observing the differences at each stage of brain development in brain architecture, synaptogenesis, neuronal differentiation, neurochemical signals, and myelinization, we can intuit that it is not possible to apply the adult concepts to children at different ages of development [14].

In human neonates, maturation of receptor activity has a critical period dependent on synaptogenesis and plasticity between birth and the age of two. Changes in the maturation of glutamate and gamma-amino butyric acid (GABA) receptors in the developing brain show that GABA activation generates significant depolarization, even months after birth. Inhibitory activity is gradually reached through development. Before full maturation of GABA-mediated inhibition, the ***N*-methyl-D-aspartate receptor** (NMDA receptor) and **α-amino-3-hydroxy-5-methyl-4-isoxazolepropionic acid receptor** (AMPA receptor) subtypes of glutamate receptors peak in the neonatal period. Kainate receptor binding is initially low and gradually rises to adult levels by the fourth postnatal week [15].

If there is a high predominance of GABAergic activity in immature brains (preterm/newborns) and given its recognized role in epileptogenic activity at this age, we should question why the extensive use of GABAergic drugs such as propofol and sevoflurane continues to be promoted in this population, considering the risk of producing an as yet not well-defined excitatory condition. This excitatory GABA state produces a kind of disappearance of a minimal “state of consciousness” or perhaps simply the state of vigilance for these developing brains [16].

Neuroapoptosis, or programmed cell death, is a physiological process of adjustment and occurs at an accelerated rate from 24 weeks of gestation until 4 weeks after birth. This process involves γ aminobutyric acid (GABA) and the glutamate neurotransmitter and receptor systems. This is a very important process for building the fetal brain connectome. In the absence of transmission and neuronal binding of GABA and glutamate, neurons produce neuroapoptosis in an unordered way. The majority of commonly used anesthetic and sedative agents bind to the GABA receptor or the N-methyl-D-aspartate receptor and affect neuroapoptosis very intensely [17]. The binding of GABA and NMDA agents blocks normal neurotransmission in the GABA and glutamate systems, resulting in synaptic deprivation and probably decreasing the formation of dendrites and disrupting neuronal migration. There is an unresolved discussion about the clinical impact of these findings, and from a clinical point of view, the focus of these investigations probably should be on repeated exposure to anesthesia, or its effect on vulnerable brains [18,19,20].

In neonates, synaptogenesis has different densities in different brain areas. In prefrontal areas, the density is less abundant and reaches its peak only 12 months after birth [21]. This may be important in the different EEG tracing in neonates consistent with the lack of thalamo-cortical synchrony seen in anesthetized adults and may be part of the deleterious consequences in the development of these areas exposed to anesthetic drugs commonly used in mature brains.

Glucose uptake in positron emission tomography (PET) studies also shows differences between different areas, with abundant and early activity in occipital zones at 3 months postnatal, while temporal activity begins at 6–8 months [22].

Subplate neurons, the first neurons generated in the cerebral cortex, play a key role in brain development, and guide the formation of thalamocortical connections. The subplate cells form the first functional connections and are necessary for relaying early oscillatory activity in the developing brain [23].

Neonates are affected by the inhibition of intrauterine wakefulness factors that disappear at birth. For example, norepinephrine reuptake is activated along with the activity of the locus coeruleus as a global amplifier center, stimulating arousal and wakefulness [24].

This could justify the use of dexmedetomidine in the newborn by creating conditions like the intrauterine stage of lower connectivity to the environment.

Baseline neuroapoptosis increased in neonatal rats exposed to isoflurane, but it was noted that it did not increase in neonatal rats exposed to dexmedetomidine alone. When dexmedetomidine was added to the isoflurane exposure group, they observed a dose-dependent reduction in neuroapoptosis to basal level with no effect on long-term cognitive outcome [25,26].

The recording of neural activities from use of dexmedetomidine show weak effects on the EEG; they appear to demonstrate incomplete elimination of synchronies such as phase–phase synchrony rhythms. However, from results in short-term studies, there were multiple strong correlations between the base state without the drug and the change of some basal feature value after the drug administration. This apparently indicates that dexmedetomidine did affect the cortical activity of the subjects, but probably indirectly [27].

There are some limitations regarding the safe use of dexmedetomidine in children with congenital heart disease because sinus node and atrioventricular nodal function are both depressed [28,29]. However, the drug is being explored for use in neonate rats that have suffered perinatal asphyxia due to its neuroprotective effect when combined with therapeutic hypothermia [30].

EEG and postnatal brain tractography show significant age differences in these patients. Synaptogenic arborization is also highly differentiable between neonates, young children, and adolescents [31].

The development of the nervous system begins in the first weeks of gestation. In this phase of brain development, the neonatal cortical activity recorded with the EEG has unique characteristics that can be differentiated from that of an adult. In children before 37 weeks of gestational age, there is a discontinuity compared to the EEG of an adult, and the premature EEG is normally classified as continuous or discontinuous. An intermittent burst of activity in the presence of an otherwise discontinuous EEG is common in premature infants. These bursts of activity are called transient spontaneous activity (TSA) [32]. They are believed to originate in the subplate area of the neonatal brain, and the cortical subplate area is believed to regulate the cortical activity of premature infants [33]. As the baby matures, the EEG becomes increasingly continuous, and the TSAs disappear. The EEG activity of a full-term infant is already continuous during wakefulness and sleep. Another difference is the expression of the stages of sleep, where after only 36 weeks, these states can be correlated with three different EEG patterns. Calm sleep is characterized by discontinuity, while active sleep is continuous [34].

From the point of view of consciousness, the fetus at birth is already aware of the body, perceiving pain and differentiating between touch and nontouch. It also presents facial expressions to certain stimuli, probably preprogrammed and of subcortical origin. It is capable of expressing emotions and shows signs of shared feelings. However, as described by Changeux, it is unthinking, present-oriented, and makes little reference to the concept of self [35]. The absence of a sense of self poses difficulties in the interpretation of how these individuals’ anesthetic–surgical experiences will be stored and subsequently incorporated into the bank of experiences.

Newborns show characteristic features of what can be called basic consciousness, and still have to undergo considerable maturation to reach the level of adult consciousness, although as a premature infant they may open their eyes and make minimal eye contact with their mother, showing avoidance reactions to harmful stimuli.

The immature brains of children under six months may be much more dependent on activating (arousal) activity than on intrinsic cortical activity, because the bank of experiential memory and the characteristics of connectivity are still extremely limited, preventing the emergence of what we define as explicit perceptual awareness.

On the other hand, in adults, propofol induces a state of unconsciousness with an important alpha activity in the frontal spectrogram, resulting from the synchronic thalamo-cortical activity [36].

**Figure 1:**
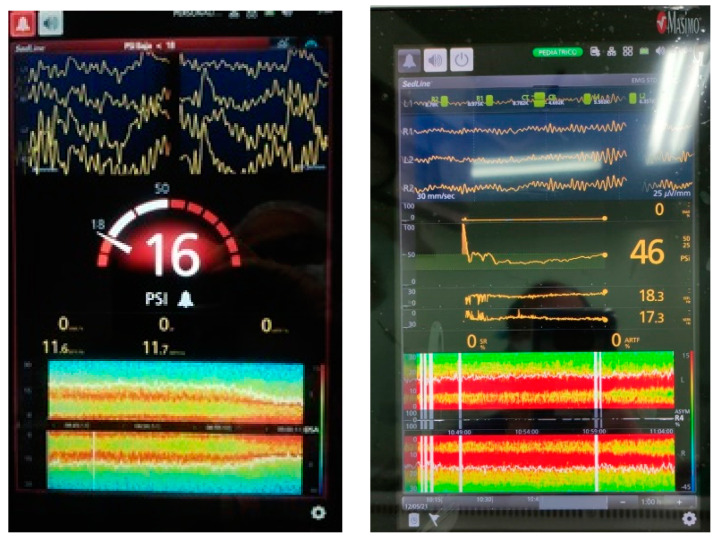
Up Left: Raw EEG and spectrogram from a 45-year-old patient receiving a typical Propofol anesthesia. Down at the end of the screen, observe the effect of adding Sevoflurane. Right: A 10-year-old child’s raw EEG and spectrogram. The global power and the alfa band (7–12 Hz) is much intense than in adults. (Sedline Monitor).

**Figure 2:**
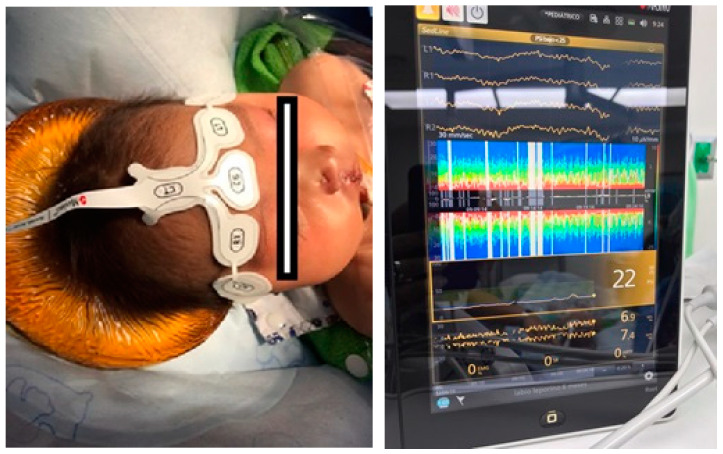
A 6-month-old patient. The EEG shows a spectrogram with very low potency in the alfa band despite low concentrations of propofol (1.6 ug/ml).

This frontal delta spindle activity is not observed in children under 6 months. It appears only in children and adolescents where the overall activity (power) is much more intense than in adults. This is seen in the intensity of these alpha bands (Figure 1). In children under 6 months, the mechanism for frontal predominance of alpha power suggests that the differential thalamic connectivity required to produce this phenomenon is not present (Figure 2). A study examining the EEG effects of sevoflurane during induction, maintenance, and awakening revealed delta oscillations at all ages; but the theta and alpha oscillations began to appear only at 4 months. As commented, in infants both frontal alpha and coherence are missing, and upon awakening theta and alpha activity begins to decrease only from 4 to 6 months onwards [37].

Brain monitoring during anesthesia with EEG-derived indices is used only to a limited extent in adults, rarely in children, and essentially not at all in infants. These EEG-derived indices, which have been developed in adults, can give inaccurate indications of anesthetic states in infants and younger children [38,39].

The current pediatric research has studied a limited set of anesthetics and has frequently used EEG montages with few electrodes. Different analysis methods have been used in different studies. Multielectrode recordings available in children have not been analyzed in relation to age. Dose-titration experiments commonly conducted in adult patients cannot be conducted in children for ethical reasons [38,40].

The EEG has characteristics that consistently change with arousal during anesthesia, but the relationship between arousal and the EEG is imprecise and drug dependent. This relationship is the basis for using the EEG to measure anesthesia and provides only an indirect measure of consciousness and memory formation in adult patients.

Given the mentioned physiology in developing brains, the cortical activity is highly dependent on the activating ascending system (arousal) required to sustain an active level of consciousness, and it is minimally dependent on the contents of consciousness. In clinical pharmacologic terms, this means that drugs with subcortical predominant effect, such as opioids, alpha-2 agonists, or low doses of ketamine, are more fundamental than the use of GABAergic drugs to depress the level of consciousness, to simply reduce the arousal and put the cortex in a more synchronic rhythm.

In older children, the problem is different. Data indicate that the brain is continually active and the EEG during loss of consciousness shows broad and very powerful alpha bands. The great challenge from the clinical point of view is the phenomenon of emergence delirium. To date, the causes have been poorly explained, but it is likely that this phenomenon is produced by a transitory alteration of the connectivity together with an overload of neuronal input coming from the surgical stimulation or from the postoperative environment. This data overload on a pharmacologically affected brain would generate an information-integration imbalance. Data demonstrating the excitation of the locus coeruleus by the effect of sevoflurane may explain this “overamplification” effect due to the norepinephrine released in this circumstance and eventually may explain emergence delirium in children [41]. An imbalance in the ability to process and integrate information in frail, underdeveloped brains or those affected by drugs would explain the frequent phenomenon of postoperative excitation or delirium.

## 4. Pharmacology

Available pharmacological data on children are also weak. The formulas describing distribution and elimination are complex, and weight as a covariable becomes insufficient to describe change over a wide range of ages and sizes. The concept of allometry has been incorporated into pharmacokinetics/pharmacodynamics (PK/PD) models to correct for the change in function and size across ages, particularly in relation to clearance. This approach (use of allometry and other covariables in modeling) help to explain that weight has a different impact for each distributive phase or elimination clearance. It is not correct to use a single working weight to calculate the dose during all the anesthesia, but several, as the distributive process progresses [42,43].

Inhaled drugs have been the most widely used in neonatal population, but clinical data are primarily extrapolations from older children and based on the concept of immobility (MAC). To study the MAC awake (statistical partial pressure of gas to put 50% of the patient unconscious) in this population is very questionable because of the lack of definition of consciousness in this population and absence of EEG correlates of these clinical states [44].

In the case of dexmedetomidine the PK models with significant casuistry are only found in children over 2 years [45].

In a recent study with 20 term infants, the most suitable model for dexmedetomidine was a mono-compartment model with an allometric clearance adjusted to postmenstrual age (PMA). PMA is defined as the time (in weeks) elapsed between the first day of the last menstrual period and birth (gestational age) plus the time elapsed after birth (chronological age). Currently there is no validated and approved target-controlled infusion technology available for clinical use to optimize the administration [46].

A study with intensive care pediatric patients using four different data sets observed that the sedation concentration was similar to adults, but small children under 1 year of age with immature clearance required a dose adjustment [47]. 

The studies of propofol, despite having managed to describe a broad spectrum of ages from infants to adolescents [48,49,50], have shown that in young children, the initial distribution volumes are oversized, generating an initial overdose [51,52].

**Figure 3:**
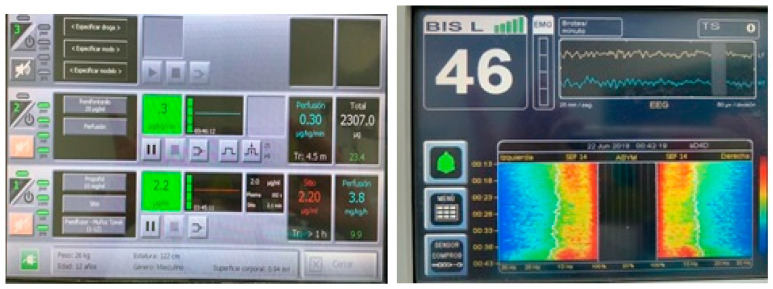
A typical overdosing of a 12-year-old patient using Propofol children model (Paedfusor) in TCI mode. You can observe a depression in the alfa band in the middle of the spectrogram display (Time is from up to down). This depression is considered a sign of overdosing and requires the reduction of the concentration of propofol observed at the down point of the screen (BIS bilateral monitor left, Ezfusor™ infusion system right). The adequate doses are only 2.2 ug/ml in this case.

In our experience, the most appropriate model to use is Paedfusor (Absalom) [51], because it includes covariates from 2 months of age to advanced adolescents (Figure 3). The Kataria [48] model only included children between the ages of 3 and 9. The ideal way to overcome this initial overdose would be to perform 2 min stepwise titrations until clinical unconsciousness is achieved. This procedure unfortunately involves having previously placed a venous line. In all these cases, the use of reference PK/PD models require target-controlled infusion technology, which is still not available in the USA, but is used throughout South America, Europe, and Asia.

Pharmacodynamic models in children also have weaknesses. In PK/PD models, the unifying link between the dissociation of plasma concentrations and the appearance of the effect is called ke0. This ke0 is a constant of gradient proportionality that describes the time course of the effect and has been of great help in the possibility of titrating adult patients during inductions or moments of changes of anesthetic requirement. However, it needs to be refined in consideration of the phenomenon recently described as neuronal inertia. This protective phylogenetic phenomenon is necessary to maintain bistable states and is not represented in the reference studies of concentration–effect relationships used in current pharmacology [53,54].

As commented regarding the changes in the EEG, the biological markers that were used in children (bispectral index (BIS) monitor, spectral edge frequency (SEF50), or auditory evoked potential (AEP)) for the construction of these models, do not represent recent advancements in neuroscience. For example, in a Chilean study with children from 3 to 11 years of age, the mean ± standard deviation of the time to peak effect (TTpeak) of the BIS index was 65 ± 14 s and 201 ± 74 s with the Alaris AEP index. Validation of the effect model and its ke0 was only performed with the BIS monitor. In this case, the use of TTpeak is questionable because it is based on measurements made after a manual bolus where conditions of equilibrium between the plasma and the biophase cannot be ensured [55]. 

For now, what is becoming clear is that children have faster equilibrium times than adults [56,57].

Another study with Chilean children and adults compared the calculated plasma concentrations (Cp) using the pharmacokinetic models of Kataria [48] and Marsh [49] to reach a BIS of 50. This study showed that the Cp to achieve BIS 50 were similar, concluding that the difference between children and adults is essentially pharmacokinetic. From the current perspective, this study is biased because it assumed that the BIS algorithm works equally in both populations, a fact that we currently know is not the case.

The problem is even more significant in children under one year of age, for whom the PKPD data are even weaker. Questions arise here as to whether the use of GABAergic drugs in these brains that still have high excitatory GABA activity produces an anesthetic state, or perhaps just an epileptiform excitatory connectivity uncoupling. That is one of the reasons why the discussion of the potential synaptogenesis alterations that may impact late on cognitive performance is taking place. In fact, we could consider that in neonates up to 6 months, whose thalamo-cortical tracts are poorly developed, the emergence of experiential perceptual consciousness can even be discussed due to the difficulty of producing the phenomenon of “integration of information for complexity” [58] due to the type of cortical activity at that age, the minimal load of stored memory, and what is preferably dependent on subcortical emotionality without explicit memory (the concept of the proto-self is developed by Antonio Damasio in his book *How the Brain Creates the Mind*, 2010).

Currently, it is very uncommon to use any type of neuromonitoring in children, but there are studies that have reported that isoelectric EEG events were common in infants and young children undergoing sevoflurane or propofol anesthesia. The dosing, based frequently on patient hemodynamics and weak data coming from population pharmacokinetics, is often associated with unnecessarily deep anesthesia during surgery [59].

In a recent study in children 0–37 months of age requiring general anesthesia for noncardiac or intracranial surgery using sevoflurane or propofol infusion, in 63% of the patients, an episode of isoelectricity was observed, more related to high ASA status patients [60].

**Figure 4:**
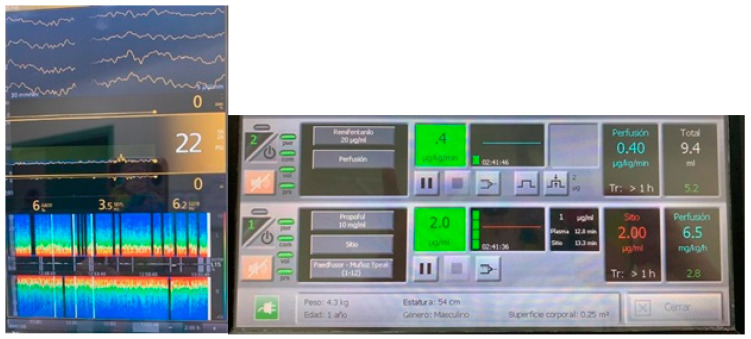
A 6-weeks old child, 4.3kg, biliary atresia, General anesthesia with Propofol TCI, Remifentanil manual calculation. The raw EEG and the spectrogram show an important depression of the SEF95, many Burst suppression episodes and asymmetry between both hemispheres, no alpha activity using extremely low Propofol concentration.

The true predictive value of the occurrence of burst suppression in pediatric anesthesia remains unknown. Still, it suggests that dosing based on population pharmacokinetics and patient hemodynamics is often associated with unnecessarily deep anesthesia during surgical procedures. There is a paucity of data on the incidence of overdose due to the low use of EEG monitoring in this population, but we suspect that overdose must be a common phenomenon due to the way in which the practice of sedation/unconsciousness titration in pediatric anesthesia is performed. (Figure 4)

There is widespread discussion on how to address all these challenges in a multifactorial way to reflect whether current anesthesia practice has a deleterious impact on pediatric patient outcomes [61].

In summary, recent data in young children indicate that anesthetic drugs that act by GABAergic mechanisms and NMDA produce a greater risk of generating alterations in maturation. This would be more evident at repeated doses.

The use of anesthetic concepts based on clinical and EEG definitions of how mature brains respond does not work for developing brains. Knowledge in these areas is still limited. 

In clinical practice, it is exceptional to finely titrate drugs based on well-defined objectives, precisely because they are weak; for example, the clinical definition of unconsciousness, or if cortical-thalamic-cortical uncoupling is required as in adults to obtain a neuronal depression that has the minimum impact on brain development.

The deficit in drug modeling and not yet having automated administration technology available in target-controlled infusion (TCI) are complexities to be addressed.

A better understanding of the EEG activity required to generate brain protection, avoiding isoelectric or convulsive phenomena, and identifying the EEG condition associated with a potential implicit memory of the surgical experience are now on the list of knowledge and concepts that must be progressed.

## Data Availability

Non applicable.
